# Supplementary material for: Effectiveness and Safety of Ayurvedic Medicines in Type 2 Diabetes Mellitus Management: A Systematic Review and Meta-Analysis
Source: Front Pharmacol. 2022 Jun 8;13:821810. doi: 10.3389/fphar.2022.821810 (PMC9213670; doi:10.3389/fphar.2022.821810)
Supplement: Supplementary file 1 [file DataSheet1.zip › Supplementary Material/Forest and Funnel Plots/Cinnamomum aromaticum/Funnel plots/Funnel plot_Cinnamomum aromaticum_HbA1c.pdf]

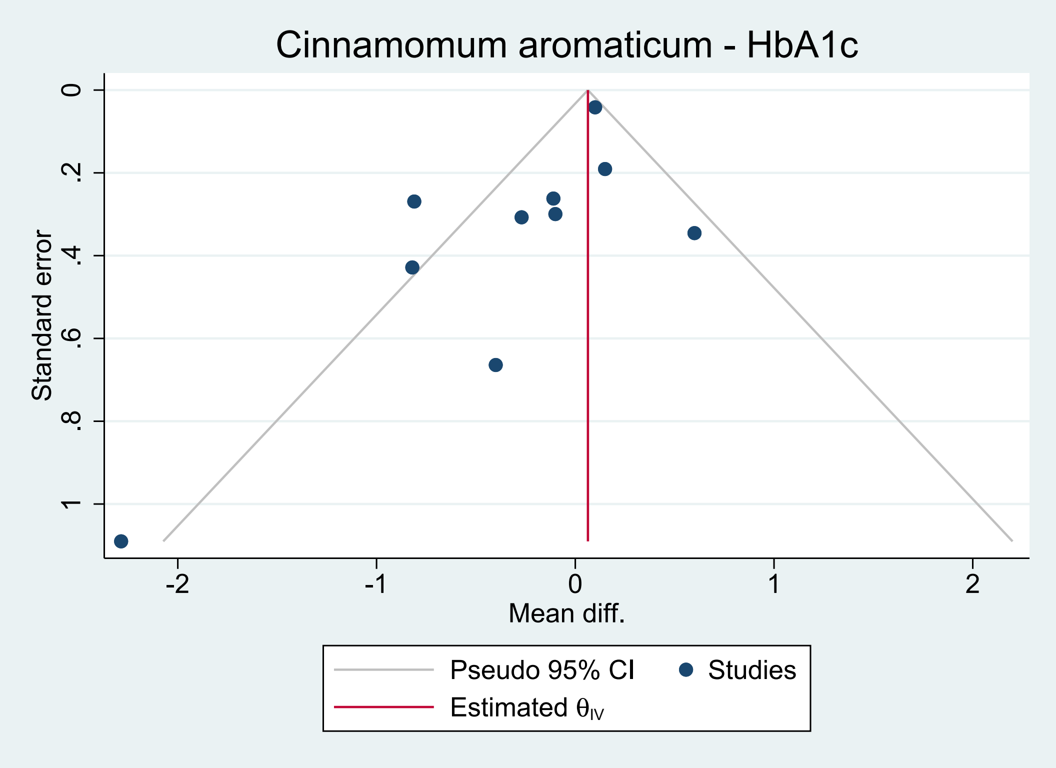

Cinnamomum aromaticum - HbA1c
Standard error
0
.2
.4
.6
.8
1
-2
-1
0
1
2
Mean diff.
Pseudo 95% CI
Studies
Estimated θ_{IV}
